# John C. H. Spence (1946–2021)

**DOI:** 10.1107/S2052252521007879

**Published:** 2021-08-19

**Authors:** Henry N. Chapman, S. Samar Hasnain, Uwe Weierstall

**Affiliations:** a CFEL, Deutsches Elektronen-Synchrotron DESY, Notkestrasse 85, Hamburg, 22607, Germany; b Institute of Systems, Molecular and Integrative Biology, University of Liverpool, Life Sciences Building, Crown Street, Liverpool, Merseyside L69 7ZB, United Kingdom; c Arizona State University, PO Box 871504, Tempe, AZ 85287, USA

**Keywords:** obituary, X-ray imaging, high-resolution electron microscopy, serial femtosecond crystallography, X-ray free-electron lasers

## Abstract

Obituary for John C. H. Spence.


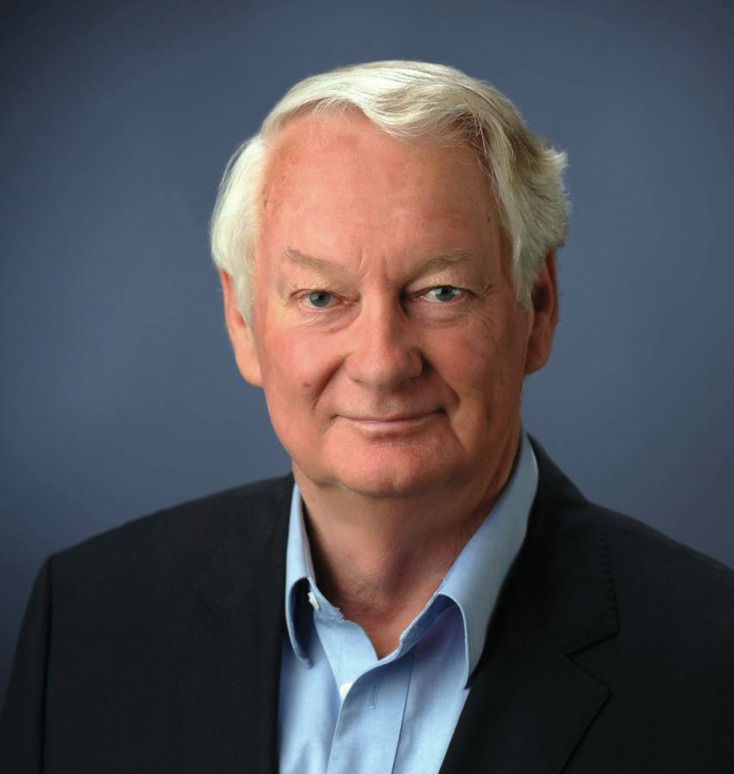


John C. H. Spence ForMemRS, Regent’s Professor and the Richard Snell Professor of Physics at Arizona State University (ASU), who continuously blazed thrilling new trails in the science and methodology of electron and X-ray imaging, died on 28 June 2021. John’s career spanned developments in high-resolution electron microscopies to make the first observations of dislocation kinks at the atomic scale and the bonds in metal oxides and GaAs, to projection imaging using electrons emitted from a single-atom field-emission tip, to some of the first demonstrations of lensless X-ray imaging, and culminating in the spectacular new field of serial femtosecond crystallography using X-ray free-electron lasers. He was fascinated by the phase problem in diffraction and crystallography, with a close intellectual connection to Lawrence Bragg who made first attempts to utilize changes in unit-cell dimensions to map out the underlying molecular transform. The brightness inherent to undulator devices at synchrotron radiation facilities and, later, to X-ray free-electron lasers, provided coherent beams and fertile ground for fresh new ideas and methods in imaging and diffraction. John’s sweeping knowledge of Fourier optics and microscopy theory, coherent micro-diffraction, dynamical diffraction, solid-state and quantum physics, as well as an inventiveness for instrumentation harking back to the age of enlightenment (some of his heroes were Lord Rayleigh, Humphry Davy and James Clerk Maxwell) brought forth a stream of new instruments and methods. John was a prodigious writer of papers, books, emails, lists and post-it notes. He shared ideas freely and widely through such writings and through his enthusiastic talks and interactions with colleagues and strangers all around the world.

John was born in Canberra, Australia. His father was a decorated Royal Australian Air Force (RAAF) pilot who was killed in action in the Korean War when John was four years old. His mother, a strong personality, imbued him with a keen sense of fairness, empathy, humanism and kindness that set him apart over the course of his entire academic career. He carried out his PhD at Melbourne University with Alan Spargo on electron energy loss spectroscopy (EELS). Together with CSIRO, Melbourne University was an early bastion of electron microscopy. In a postdoctoral fellowship at Oxford University with Sir Peter Hirsch, David Cockayne and Mike Whelan, John advanced dynamical diffraction theory and the pursuit of imaging atoms in the TEM, using channelling and lensing effects caused by columns of atoms in a crystal. He arrived in the USA in 1976 to join the group of John Cowley, a former Melbourne professor and founder of the Center for High Resolution Electron Microscopy at Arizona State University. There, he worked on the theory of the scanning transmission electron microscope (STEM), continued EELS in this microscope configuration, and further developed ideas to use the electron wavefield propagating through a crystal as a form of lens or as a way to locate dopant atoms by measuring X-ray fluorescence as a function of the phase of standing wave. He used high-resolution TEM to image defects in semiconductors, solving the famous question of the ‘shuffle or glide’ mechanism of dislocation cores in silicon. In 1995, together with Harry Kolar he was able to image moving dislocation kinks along partial dislocations by taking advantage of forbidden f.c.c. reflections. He also made the first observations of *Pendellösung* radiation and coherent *Bremsstrahlung* due to electrons passing through the periodic potentials of crystals, a forerunner of his latest work at the BioDesign Institute, together with Bill Graves, on developing a compact XFEL.

In the 1980s, as an assistant professor and together with Mike O’Keefe and Jian-Min Zuo, John continued developing convergent-beam electron diffraction in the STEM to quantitatively and accurately map out charge density distributions. This culminated in the direct observation of atomic *d*-orbitals of copper oxide. With Mike O’Keefe in 2005, he also was the first to apply the charge flipping algorithm to experimental data and solve a real structure. At this time, John also started to investigate various mathematical approaches to directly solve the many-beam dynamical diffraction inversion problem for determining unknown crystal structures. He kept returning to this quest for the rest of his life and was finally able to publish an elegant and complete solution based upon an iterative method of projections together with Jeffrey Donatelli. In a talk he gave on the topic in May 2021, John remarked on how satisfied he was to have completed this work, saying it was what he was most proud of. But in addition to these theoretical underpinnings, John was quite the tinkerer and had a love of instrumentation (and vintage cars). One major achievement coming out of John’s lab was the first CCD camera for electron microscopy in 1986, later fully developed by Gatan. In the early 1990s, he was able to obtain low energy electron projection and reflection holograms using a single atom electron field emission source, with Weida Qian and later Uwe Weierstall. Around that time John became interested in and investigated the possibility of imaging biological specimens at electron energies below the carbon *K*-shell to reduce radiation damage. With Theo Vecchione, he worked on designing a coherent photofield electron source for fast imaging. Enthralled by the invention of the scanning tunnelling microscope, he placed one inside a TEM holder, together with William Lo, to make simultaneous STM and reflection mode TEM images. He then combined, together with Uwe Weierstall, an STM with a time-of-flight spectrometer for identifying atomic species on surfaces. He often quoted Humphry Davy, ‘Nothing tends so much to the advancement of knowledge as the application of a new instrument’ and loved the ingenuity of our scientific forebears (his joy jumps off the pages of his recent book ‘Lightspeed’ on the history of the measurement of the speed of light).

In addition to his developments of electron microscopy for materials, John was the driving force to obtain an NSF grant to acquire a cryo-EM at ASU, which is now installed in the Southwestern Center for Aberration Corrected Electron Microscopy. For his achievements in Electron Microscopy, John received the Distinguished Scientist Award of the Microscopy Society of America in 2006, the Buerger Medal of the American Crystallographic Society in 2012, the J. M. Cowley Medal of the International Federation of Societies of Microscopy in 2014, the Burton Medal of Microscopy Society of America (1980) and a Humboldt Senior Scientist Award in 1991. John became a Fellow of the Royal Society (Foreign Member) in 2015, and a Corresponding (Foreign) Member of the Australian Academy of Science in 2016. He was also a Fellow of the American Association for the Advancement of Science, of the National Academy of Inventors, the American Physical Society, the Microscopy Society of America, of the Royal Microscopy Society, of the Institute of Physics (UK), and an Overseas fellow of Churchill College Cambridge, UK.

A sabbatical at Lawrence Berkeley Laboratory in 1997–1998 and a decade of summers spent there in research and on scientific advisory committees brought about a new chapter in John’s interests. The demonstration of direct phasing of a coherent X-ray diffraction pattern of a non-periodic object by John Miao, David Sayre and others in 1999, sparked John’s interest. He initiated a long-running series of international workshops on coherence and phase retrieval where he brought together the communities of convergent-beam electron diffraction, electron holography, electron ptychography, the budding X-ray diffractive imaging, and leading experts in non-convex optimization problems. John began investigating the opportunities and limits of the approach with Malcolm Howells (Henry Chapman later joining). They carried out the first demonstration of lensless imaging in three dimensions, using soft X rays, and explored limitations due to radiation damage. A question was whether the approach could realize atomic imaging of macromolecules without needing to crystallize them. While the idea of out-running radiation damage using would-be free-electron laser pulses had just been published by Janos Hajdu’s group, John had many other ideas which he pursued with characteristic vigour, such as diffracting with a continuous X-ray or electron source from a stream of molecules aligned in a polarized laser field, and resurrecting an old idea, due to Kam, of calculating intensity correlations from snapshot coherent diffraction patterns of an ensemble of identical but randomly oriented molecules. Correlations of photons scattered from the same molecule would accumulate whereas those from different molecules would wash out. The short exposure times would freeze out any blurring due to rigid-body rotations. John coined the phrase ‘diffraction before destruction’ for outrunning the Coulomb explosion with intense femtosecond-duration pulses and joined the large collaboration, along with Janos Hajdu, Ilme Schlichting, Petra Fromme and Henry Chapman and many others, to carry out first experiments at the Linac Coherent Light Source, the first X-ray FEL. The success of the method which was later termed serial femtosecond crystallography (in homage to John’s earlier idea of creating orientational order with a laser) relied on many factors – one of the most important being liquid microjets for crystal delivery, that John perfected with Bruce Doak, Dan Deponte and Uwe Weierstall. The method became the most widely used technique at XFELs, providing radiation-damage-free structures of macromolecules at room temperature and with temporal resolution. This was in no small part due to the effort of many collaborators in the NSF Science and Technology Center ‘BioXFEL’ which John founded and of which he was Director of Science, and which now consists of 13 partner institutions. He was always open to new ideas and generous with his time, encouraging new projects and researchers wishing to make use of the new XFEL technology. Thus he paved the way of utilizing serial crystallographic approaches to obtain structures free from radiation-induced chemistry (and associated structural changes), and structural enzymology via developments such as mix-and-inject systems. Together with Janos Hajdu and Henry Chapman, John was awarded the 2021 Gregori Aminoff Prize of the Royal Swedish Academy of Sciences for their contributions to FEL-based structural biology. Sadly, John did not live to attend the ceremony which was postponed due to the current pandemic.

John was a prolific writer and his monographs *High Resolution Electron Microscopy* and *Electron Microdiffraction* (with Jian-Min Zuo) are indispensable classics for electron microscopists. His emails, like his conversations, were full of irreverent wit, blinding insights, advice and encouragements, and often hard to keep up with. His writings showed in-depth understanding of the topic but clear understanding of the factors that contributed to the success of the latest advancement. One such example is his first editorial, entitled ‘X-ray lasers and crystallography’, as the Main Editor (Physics and FEL Science and Technology) of **IUCrJ** where he wrote ‘As so often happens in the history of science, the breakthrough eventually occurred at the interface of several fields – synchrotron science (and especially their insertion devices), laser physics, and work on microwave tubes for radar, emerging from the second world war.’ ‘All of this culminated recently in the construction of the first hard-X-ray laser, the US Department of Energy’s Linac Coherent Light Source (LCLS), at their SLAC laboratory near Stanford. The first X-ray lasing occurred in that two-mile long tunnel on 21 April 21 2009 at about 2 kV, in an all-or-nothing moment of intense excitement, as theoretical predictions proved spot-on.’ He went on to acknowledge foresight of DOE ‘The gamble taken by the DOE in committing to the $600 × 10^6^ construction of the LCLS around 2004 was laudable, in these days when low-risk incremental science seems the only way to attract funding against high odds. Will it lase? Will it be useful? The past five years have seen their vision vindicated with breakthrough applications in many fields, from materials science and atomic and molecular physics to condensed matter physics and biology.’

He had a high sense of community service which was reflected in many arenas including his service to the International Union of Crystallography. In addition to his position as the Main Editor (Physics and FEL Science and Technology) of **IUCrJ** since the journal’s launch, John was Co-editor of *Acta Cryst. A* from 1989 to 1999 and served on the IUCr Commissions on Electron Diffraction (now Electron Crystallography) (1996–2002; Chair 2002–2005) and Charge, Spin and Momentum Densities (now Quantum Crystallography) (1999–2008). When in 2012, after one of us (SSH), as Editor-in-chief of the IUCr journals, approached John to lead one of the sections (Physics, and FEL Science and Technology) at the ASCA meeting at Adelaide outlining the vision and ambition for **IUCrJ**, he immediately saw its importance. Despite enormous demands on his time he agreed and started coming up with ideas how to make **IUCrJ** a natural home for FEL Science and Technology. The launch of the **IUCrJ** was announced on 5 December 2012 with five main editors (E. N. Baker, C. R. A. Catlow, G. R. Desiraju, S. Larsen, J. C. H. Spence). In addition to assembling a fantastic team of Co-editors for his section and encouraging key authors to submit high quality papers, John led from the front as is reflected in an important contribution, entitled ‘*Ab-initio* phasing using nanocrystal shape transforms with incomplete unit cells’ that appeared in the Inaugural issue.

John had many other interests included sailing, vintage cars and music. He was passionate about playing the piano, guitar and flute, and performed with other ASU faculty and postdocs in several bands over the years. His most recent ensemble ‘Who Knew’ wowed attendees of many conferences – they were a special feature of the BioXFEL conferences.

John will be remembered for his wit, generosity, openness, sense of duties to the wider community and above all for his tremendous scientific contributions across the sciences, reflected in some of the books and papers given below.

John is survived by his wife, Margaret, sister, Penny, son, Andrew with his wife Rebecka and two grandchildren, and by three stepsons, James, Daniel and Peter Stanzler and their families.

## References

[bb1] Chapman, H. N., Fromme, P., Barty, A., White, T. A., Kirian, R. A., Aquila, A., Hunter, M. S., Schulz, J., DePonte, D. P., Weierstall, U., Doak, R. B., Maia, F. R. N. C., Martin, A. V., Schlichting, I., Lomb, L., Coppola, N., Shoeman, R. L., Epp, S. W., Hartmann, R., Rolles, D., Rudenko, A., Foucar, L., Kimmel, N., Weidenspointner, G., Holl, P., Liang, M., Barthelmess, M., Caleman, C., Boutet, S., Bogan, M. J., Krzywinski, J., Bostedt, C., Bajt, S., Gumprecht, L., Rudek, B., Erk, B., Schmidt, C., Hömke, A., Reich, C., Pietschner, D., Strüder, L., Hauser, G., Gorke, H., Ullrich, J., Herrmann, S., Schaller, G., Schopper, F., Soltau, H., Kühnel, K.., Messerschmidt, M., Bozek, J. D., Hau-Riege, S. P., Frank, M., Hampton, C. Y., Sierra, R. G., Starodub, D., Williams, G. J., Hajdu, J., Timneanu, N., Seibert, M. M., Andreasson, J., Rocker, A., Jönsson, O., Svenda, M., Stern, S., Nass, K., Andritschke, R., Schröter, C.., Krasniqi, F., Bott, M., Schmidt, K. E., Wang, X., Grotjohann, I., Holton, J. M., Barends, T. R. M., Neutze, R., Marchesini, S., Fromme, R., Schorb, S., Rupp, D., Adolph, M., Gorkhover, T., Andersson, I., Hirsemann, H., Potdevin, G., Graafsma, H., Nilsson, B. & Spence, J. C. H. (2011). Femtosecond X-ray protein nanocrystallography. *Nature*, **470**, 73–77. 10.1038/nature09750 PMC3429598 21293373

[bb2] Conrad, C. E., Basu, S., James, D., Wang, D., Schaffer, A., Roy-Chowdhury, S., Zatsepin, N. A., Aquila, A., Coe, J., Gati, C., Hunter, M. S., Koglin, J. E., Kupitz, C., Nelson, G., Subramanian, G., White, T. A., Zhao, Y., Zook, J., Boutet, S., Cherezov, V., Spence, J. C. H., Fromme, R., Weierstall, U. & Fromme, P. (2015). A novel inert crystal delivery medium for serial femtosecond crystallography. *IUCrJ*, **2**, 421–430. 10.1107/S2052252515009811 PMC4491314 26177184

[bb904] Conrad, C. E., Nelson, G., Stander, N., Zatsepin, N. A., Zook, J., Zhu, L., Geiger, J., Chun, E., Kissick, D., Hilgart, M. C., Ogata, C., Ishchenko, A., Nagaratnam, N., Roy-Chowdhury, S., Coe, J., Subramanian, G., Schaffer, A., James, D., Ketwala, G., Venugopalan, N., Xu, S., Corcoran, S., Ferguson, D., Weierstall, U., Spence, J. C. H., Cherezov, V., Fromme, P., Fischetti, R. F. & Liu, W. (2017). Serial millisecond crystallography of membrane and soluble protein microcrystals using synchrotron radiation. *IUCrJ* **4**, 439–454. 10.1107/S205225251700570X PMC5571807 28875031

[bb91] Cumings, J., Zettl, A., McCartney, M. R. & Spence, J. C. H. (2002). Electron holography of field-emitting carbon nanotubes. *Phys. Rev. Lett.* **88**, 056804. 10.1103/PhysRevLett.88.056804 11863765

[bb3] Donatelli, J. J. & Spence, J. C. (2020). Inversion of many-beam Bragg intensities for phasing by iterated projections: removal of multiple scattering artifacts from diffraction data. *Phys. Rev. Lett.* **125**, 065502. 10.1103/PhysRevLett.125.065502 32845656

[bb4] Frank, M., Carlson, D. B., Hunter, M. S., Williams, G. J., Messerschmidt, M., Zatsepin, N. A., Barty, A., Benner, W. H., Chu, K., Graf, A. T., Hau-Riege, S. P., Kirian, R. A., Padeste, C., Pardini, T., Pedrini, B., Segelke, B., Seibert, M. M., Spence, J. C. H., Tsai, C.-J., Lane, S. M., Li, X.-D., Schertler, G., Boutet, S., Coleman, M. & Evans, J. E. (2014). Femtosecond X-ray diffraction from two-dimensional protein crystals. *IUCrJ*, **1**, 95–100. 10.1107/S2052252514001444 PMC4062087 25075325

[bb5] Graves, W. S., Chen, J. P. J., Fromme, P., Holl, M. R., Kirian, R., Malin, L. E., Schmidt, K. E., Spence, J. C. H., Underhill, M., Weierstall, U., Zatsepin, N. A., Zhang, C., Brown, P. D., Hong, K. H., Moncton, D. E., Nanni, E. A. & Limborg-Deprey, C. (2017). *ASU compact XFEL.* In *Proceedings of the 38th International Free-Electron Laser Conference, FEL 2017*, edited by K. Bishofberger, C. Bruce & V. R. W. Schaa, pp. 225–228, https://doi.org/10.18429/JACoW-FEL2017-TUB03. Geneva: JACoW Publishing.

[bb6] Jiang, N. & Spence, J. C. H. (2012). On the dose-rate threshold of beam damage in TEM. *Ultramicroscopy*, **113**, 77–82.

[bb903] Kirian, R. A., Schmidt, K. E., Wang, X., Doak, R. B. & Spence, J. C. H. (2011). Signal, noise, and resolution in correlated fluctuations from snapshot small-angle X-ray scattering. *Phys. Rev. E*, **84**, 011921. 10.1103/PhysRevE.84.011921 21867227

[bb7] Kupitz, C., Basu, S., Grotjohann, I., Fromme, R., Zatsepin, N. A., Rendek, K. N., Hunter, M. S., Shoeman, R. L., White, T. A., Wang, D., James, D., Yang, J.., Cobb, D. E., Reeder, B., Sierra, R. G., Liu, H., Barty, A., Aquila, A. L., Deponte, D., Kirian, R. A., Bari, S., Bergkamp, J. J., Beyerlein, K. R., Bogan, M. J., Caleman, C., Chao, T.., Conrad, C. E., Davis, K. M., Fleckenstein, H., Galli, L., Hau-Riege, S. P., Kassemeyer, S., Laksmono, H., Liang, M., Lomb, L., Marchesini, S., Martin, A. V., Messerschmidt, M., Milathianaki, D., Nass, K., Ros, A., Roy-Chowdhury, S., Schmidt, K., Seibert, M., Steinbrener, J., Stellato, F., Yan, L., Yoon, C., Moore, T. A., Moore, A. L., Pushkar, Y., Williams, G. J., Boutet, S., Doak, R. B., Weierstall, U., Frank, M., Chapman, H. N., Spence, J. C. H. & Fromme, P. (2014). Serial time-resolved crystallography of photosystem II using a femtosecond X-ray laser. *Nature*, **513**, 261–265. 10.1038/nature13453 PMC4821544 25043005

[bb8] Marchesini, S., He, H., Chapman, H. N., Hau-Riege, S. P., Noy, A., Howells, M. R., Weierstall, U. & Spence, J. C. H. (2003). X-ray image reconstruction from a diffraction pattern alone. *Phys. Rev. B*, **68**, 140101.

[bb90] Nelson, G., Kirian, R. A., Weierstall, U., Zatsepin, N. A., Faragó, T., Baumbach, T., Wilde, F., Niesler, F. B. P., Zimmer, B., Ishigami, I., Hikita, M., Bajt, S., Yeh, S.., Rousseau, D. L., Chapman, H. N., Spence, J. C. H. & Heymann, M. (2016). Three-dimensional-printed gas dynamic virtual nozzles for X-ray laser sample delivery. *Opt. Express*, **24**, 11515. 10.1364/OE.24.011515 PMC5025224 27410079

[bb9] Spence, J. C. H. (1988). *Experimental High-Resolution Electron Microscopy.* Oxford University Press.

[bb902] Spence, J. C. H. (1998). Direct inversion of dynamical electron diffraction patterns to structure factors. *Acta Cryst.* A**54**, 7–18.

[bb10] Spence, J. C. H. (1999). The future of atomic resolution electron microscopy for materials science. *Mater. Sci. Eng. Rep.* **26**, 1–49.

[bb11] Spence, J. C. H. (2017). XFELs for structure and dynamics in biology. *IUCrJ*, **4**, 322–339. 10.1107/S2052252517005760 PMC5571796 28875020

[bb12] Spence, J. C. H. (2019). *Lightspeed: The Ghostly Aether and the Race to Measure the Speed of Light.* Oxford University Press.

[bb13] Spence, J. C. H. (2021). *Spitfire Pilot – Lou Spence: A Story of Bravery, Leadership and Love.* Melbourne: Brolga Publishing.

[bb14] Spence, J. C. H. & Doak, R. B. (2004). Single molecule diffraction. *Phys. Rev. Lett.* **92**, 198102. 10.1103/PhysRevLett.92.198102 15169448

[bb15] Spence, J. C. H., Kolar, H. R., Hembree, G., Humphreys, C. J., Barnard, J., Datta, R., Koch, C., Ross, F. M. & Justo, J. F. (2006). Imaging dislocation cores – way forward. *Philos. Mag.* **86**, 4781–4796.

[bb16] Spence, J. C. H., Qian, W. & Silverman, M. P. (1994). Electron source brightness and degeneracy from Fresnel fringes in field emission point projection microscopy. *J. Vac. Sci. Technol. A*, **12**, 2.

[bb17] Spence, J. C. H., Weierstall, U., Fricke, T. T., Glaeser, R. M. & Downing, K. H. (2003). Three-dimensional diffractive imaging for crystalline monolayers with one-dimensional compact support. *J. Struct. Biol.* **144**, 209–218. 10.1016/j.jsb.2003.09.019 14643223

[bb18] Weierstall, U., Chen, Q., Spence, J. C. H., Howells, M. R., Isaacson, M. & Panepucci, R. R. (2002). Image reconstruction from electron and X-ray diffraction patterns using iterative algorithms: experiment and simulation. *Ultramicroscopy*, **90**, 171–195. 10.1016/s0304-3991(01)00134-6 11942636

[bb19] Weierstall, U., Doak, R. B., Spence, J. C. H., Starodub, D., Shapiro, D., Kennedy, P., Warner, J., Hembree, G. G., Fromme, P. & Chapman, H. N. (2008). Droplet streams for serial crystallography of proteins. *Exp. Fluids*, **44**, 675–689.

[bb20] Wu, J. S., Spence, J. C. H., O’Keeffe, M. & Groy, T. L. (2004). Application of a modified Oszlányi and Süto *ab initio* charge-flipping algorithm to experimental data. *Acta Cryst.* A**60**, 326–330. 10.1107/S0108767304012231 15218212

[bb21] Zuo, J. M., Kim, M., O’Keeffe, M. & Spence, J. C. H. (1999). Direct observation of *d*-orbital holes and Cu–Cu bonding in Cu_2_O. *Nature*, **401**, 49–52.

[bb22] Zuo, J. M. & Spence, J. C. H. (1992). *Electron Microdiffraction.* New York: Springer.

